# Effects of Propolis Supplementation on Metabolic Syndrome Indices, Isfahan, Iran, 2023: A Randomized Controlled Clinical Trial

**DOI:** 10.1002/hsr2.71499

**Published:** 2025-11-13

**Authors:** Zeinab Gholami, Mohammad Reza Maracy, Mohammad Reza Abbaspoor, Zamzam Paknahad

**Affiliations:** ^1^ Department of Clinical Nutrition, School of Nutrition and Food Science, Students′ Research Committee Isfahan University of Medical Sciences Isfahan Iran; ^2^ Department of Epidemiology and Biostatistics, School of Health Isfahan University of Medical Sciences Isfahan Iran; ^3^ School of Pharmacy Mashhad University of Medical Sciences Mashhad Iran; ^4^ Department of Clinical Nutrition, Faculty of Nutrition and Food Science Isfahan University of Medical Sciences Isfahan Iran

**Keywords:** anthropometric indices, blood pressure, fasting blood sugar, metabolic syndrome, MIND diet, propolis

## Abstract

**Background and Aim:**

Propolis is a resinous substance collected from plant buds and secretions by bees, possesses numerous medicinal and biological properties, including immune modulation, anticancer effects, antimicrobial activity, anti‐inflammatory properties, and antioxidant properties. This study aims to examine the effect of the propolis on metabolic syndrome (MetS) indices among patients with MetS.

**Methods:**

This study as a randomized controlled clinical trial (RCT), was conducted on patients with MetS who were referred to the Hazrat Ali Health Center in Isfahan. Fifty six eligible patients were classified into two groups. including MIND (The Mediterranean‐DASH Intervention for Neurodegenerative Delay) diet + placebo and MIND diet + propolis. We evaluated the MetS indices (Fasting Blood Sugar (FBS), waist circumference (WC), systolic blood pressure (SBP), diastolic blood pressure (DBP), triglyceride (TG), and high‐density lipoprotein cholesterol (HDL‐C)). We used the Shapiro‐Wilk test to determine if the distribution of quantitative variables was normal. Quantitative variables were presented as either the mean with standard deviation (SD).

**Results:**

MIND + propolis group compared to MIND group showed nonsignificant decrease about body mass index (BMI) after adjusting variables 0.9 times (10%), 0.8 times (20%), respectively, and also showed nonsignificant decrease about TG by 0.9 times (10%), and also nonsignificant increase about WC and mean arterial pressure (MAP = [SBP + 2DBP]/3) by 1.03 times (3%) and 1.3 times (30%), respectively, and HDL‐C significant increased by 12.8 times.

Registration: The current study′s procedure received approval from the Iranian Registry of Clinical Trials (www.irct.ir) on 3/28/2023, with the registration reference IRCT20230105057054N1.

**Conclusion:**

MIND + propolis group compared to MIND group showed significant increase HDL‐C. therefore; propolis increase significantly HDL‐C.

## 1 Background

MetS is becoming a significant public health concern worldwide. The increase in urbanization, high intake of energy, high prevalence of obesity, and reduced physical activity (PA) are all contributing to this issue [1]. MetS is associated with an elevated risk of cardiovascular disease and type 2 diabetes due to metabolic abnormalities. According to the National Cholesterol Education Program (NCEP) Adult Treatment Panel III (ATP III) guidelines, having three or more of the following factors ‐ low HDL‐C, central obesity, hypertension, elevated FBS, and hypertriglyceridemia considered as MetS. The high prevalence of MetS is primarily attributed to obesity [1, 2, 3, 4].

The occurrence of MetS increases significantly with age in all subgroups [3]. MetS is found in about 25% of all adults, with a higher occurrence in older age groups [5]. The prevalence of MetS is 19% among individuals aged 20–39, rising to 48% among those aged 60 and above [3]. MetS is commonly reported to affect about 30.4% of the population. Numerous studies have suggested that Approximately one‐third of the Iranian adult population aged 20 years and older is affected by MetS [6]. The prevalence of obesity in the Iranian population may be linked to their dietary patterns, such as the intake of refined carbohydrates and high‐calorie foods [7].

Prior research has demonstrated the efficacy of supplements, including vitamin D [8] and blueberry supplementation [9], in the treatment of MetS in subjects. Propolis is a substance collected from plant buds and secretions by bees, and it is then processed with saliva and bee enzymes to become a compound and adhesive material used within the hive [10, 11]. In recent years, propolis has been concerned by researchers because of its various biological and medicinal properties, including its ability to modulate the immune system, its antitumor effects, its antimicrobial and anti‐inflammatory properties, antiviral, as well as its antioxidant activity [12]. Several investigations have indicated a notable decrease in serum lipid profiles, fasting glucose levels, insulin resistance, insulin, hemoglobin A1c (HbA1c), c‐reactive protein (CRP), tumor necrosis factor alpha (TNF‐α), interleukin‐6 and liver enzymes aspartate aminotransferase, alanine transaminase, after consuming propolis [10, 11]. and increase HDL‐C [11]. Another study revealed an elevation in HDL‐C and a notable reduction in TG. Nevertheless, there were no substantial alterations noted for BMI, body weight, total cholesterol, and low‐density lipoprotein cholesterol (LDL‐C) [13]. In subjects with central obesity, propolis can reduce leptin levels [14]. One meta‐analysis study indicated a noteworthy decrease in FBS following propolis intake [15]. A review study demonstrated the potential efficacy of propolis in controlling MetS and its associated markers, possibly due to its substantial antioxidant and anti‐inflammatory properties [16]. Evidence from a review article indicates that honey bee propolis contributes to the enhancement of body cell function and the reduction of diabetes‐related complications [17]. The recent study showed that propolis may help reduce waist circumference and enhance physical health and quality of life in people with this syndrome, but it did not have any notable impact on other component of MetS [18]. propolis Polyphenols have been shown to influence thermogenesis, browning, and lipid metabolism, potentially playing a role in body weight regulation [19].

The MIND diet is a combination of the (DASH) diet and Mediterranean Diet (MD) aimed at preserving cognitive function, it emphasizes the consumption of healthy food items including: green leafy vegetables, nuts, whole grains, other vegetables, berries, olive oil, seafood, poultry, beans, and wine. Unhealthy food items includes sweets, red meat, cheese, butter and margarine, fast food, and fried foods [20].

In this study, we hypothesized that propolis supplementation would improve FBS, serum TG, serum HDL‐C, blood pressure, and WC in patients with MetS. Accordingly, the research question was: Does propolis supplementation affect FBS, serum TG, serum HDL‐C, blood pressure, and WC in patients with MetS? The primary aim of the study was to evaluate the mean levels of FBS, serum TG, serum HDL‐C, blood pressure, and WC in patients with MetS before and after propolis supplementation. The public health impact of chronic conditions such as MetS has been well‐established. Due to the limited and conflicting research on this topic, we chose to investigate the effects of propolis supplementation on MetS indicators, including waist circumference, blood pressure, blood sugar, and lipid profile in patients with MetS. This decision was influenced by the role that propolis supplementation plays in correcting MetS indices.

## 2 Materials and Methods

### 2.1 Study Design

This study is a parallel RCT, and we follow the Consolidated Standards of Reporting Trials (CONSORT(checklist by Schulz KF and et al. for the CONSORT Group. It outlines the updated guidelines for reporting parallel group randomized trials in the CONSORT 2010 Statement [21].

After being diagnosed with MetS, patients were selected in succession from the Hazrat Ali Health Center in Isfahan. We reached out to patients affiliated with the Center, and if they were willing volunteers and met the eligibility requirements, they were enlisted [22].

Our study began on June 16, 2023. Initially, the participants were educated about the significance of the research and the reasons for their involvement in the study. Eligible subjects received an informed consent form for the project.

### 2.2 Study Population

**Figure 1:**
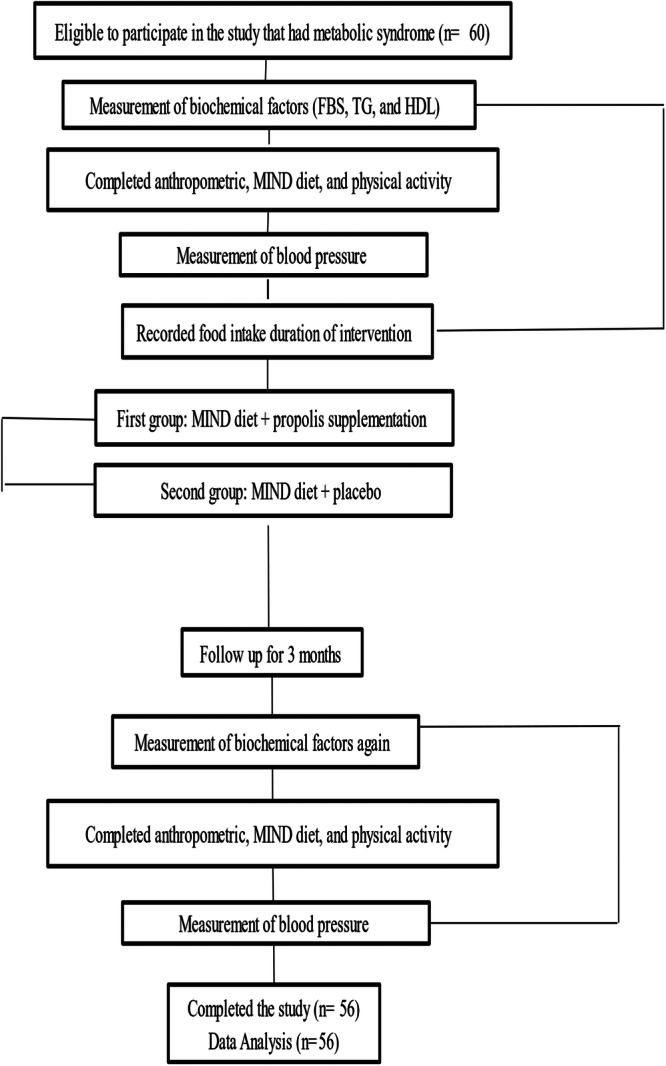
Diagram presenting the allocation and follow‐up of study participants.

This RCT conducted on patients aged 18–60 who were diagnosed with MetS according to the NCEP ATP III guidelines [23]. The study recruited 56 volunteers from Hazrat Ali Health Center in Isfahan, with 22 males and 34 females participating. Before the start of the study, two people dropped out, one because of beginning another diet, and another one was unable to continue due to trip; in such cases, replacements were quickly found for every participant who was removed from the study. The flow diagram in Figure 1 shows the progression of participants throughout the study.

### 2.3 Inclusion and Exclusion Criteria

#### 2.3.1 Inclusion Criteria

1. Participants between the ages of 18 and 60.
2. Patients who have been diagnosed with MetS based on the NCEPA ATP III criteria [23].
3. Patients with MetS who are actively seeking treatment at the Hazrat Ali Health Center in Isfahan.

#### 2.3.2 Non‐Entry Criteria

a. Had psychiatric illnesses and neurological including depression, Alzheimer's disease, Parkinson's disease, mental disorders, diabetes, anemia, thyroid disorders, and a background of receiving intensive medical treatment.
b. They had an ischemic attack or a stroke within the previous 3 months. Past medical history of human immunodeficiency virus (HIV)/hepatitis C, kidney and liver diseases, brain damage, and weight fluctuations.
c. Patients with a cancer diagnosis within the past 5 years.
d. patients who were overweight with a BMI exceeding forty.

#### 2.3.3 Exclusion Criteria of Samples

a. Patients' limited cooperation during various project stages.
b. Started another diet.
c. Became pregnant while the study was in progress.
d. They had a medical condition including memory loss, a stroke, or another ailment, that prevented them from being interviewed.
e. Weakness, severe heart failure, rheumatoid arthritis, kidney failure, chronic liver cirrhosis, inflammatory bowel disease (IBD), thyroid disease, and thinness.
f. Occurrence of blood clotting following propolis supplementation.

#### 2.3.4 Sampling

Samples were assigned to groups using a randomized block design, with patients being randomly assigned to two groups and organized in blocks of two. The aim of randomization is to minimize selection bias, which is only possible if patients giving consent cannot anticipate or influence the assignment of treatment to a patient before the trial begins. This concept is referred to as “allocation concealment” [24, 25].

There were 28 blocks of eligible patients mentioned in the study. The 1th, 2nd, and 28th blocks contain the 1th, 2nd, and 28th eligible patients, respectively. Two groups were considered: one receiving the MIND diet plus propolis and the other receiving the MIND diet plus placebo (coded as A and B). The patients for each group were assigned using a random number table, with treatment A given if the random number was 0–4 and treatment B given if it was 5–9. This allocation process can also be done using random numbers generated by a computer. After this process, the first person in each block received the corresponding treatment code based on their random number. The design expert randomly assigned numbers to treatments A and B after completing the procedure for 28 blocks. Only the designated experts knew which code corresponded to A and B at this point. The A and B codes remained undisclosed until all patients had enrolled in the study. The expert does not play a part in selecting or categorizing individuals based on personal preference or intentionally, and all steps are carried out in a completely random manner. All patients will begin the study simultaneously to prevent any errors. Moreover, the design expert does not have the responsibility of allocating treatment codes in a different section of the study design. Furthermore, the individual conducting the intervention is separate from the individual preventing bias in the data collection process.

#### 2.3.5 Information Collection Methods

We collected health history information and demographic including marital status, education level, gender, age, medical history, and use of medications, herbal remedies, and supplements through form completion and interviews. After and before the intervention, we evaluated the subjects′ blood sugar, biochemical markers, PA, blood pressure, food intake, lipid profile, and anthropometric indices.

#### 2.3.6 Anthropometrics Assessment

A qualified individual took and recorded anthropometric measurements such as WC, height, and weight. Weight was measured without shoes and with minimal clothing using a scale accurate to 100 g. Height was recorded while not wearing shoes and with a precision of half a centimeter. with the person standing against a wall with hips and shoulders touching it. The subjects' BMI was calculated: weight (kilograms)/height (meter^2^). Additionally, the WC was measured to show how fat is distributed on the body. The person being measured wore minimal clothing, and a rigid measuring tape was placed around the narrowest part between the thigh and chest without squeezing the body. The measurement was accurate to 0.01 cm [26].

#### 2.3.7 Diagnosis of Metabolic Syndrome

As per the NCEP ATP III guideline, MetS was determined to be present [23] if a minimum of three of the specified criteria were met. All participants were assessed in a dedicated laboratory at the beginning and finishing of the study.

a. TG levels ≥ 150 mg/dL.
b. HDL_C levels below 50 mg/dL for women and 40 mg/dL for men.
c. DBP ≥ 85 mmHg and SBP ≥ 130 mmHg.
d. FBS levels ≥ 100 mg/dL.
e. Men's WC ≥ 102 cm and women's WC ≥ 88 cm [27, 28].

#### 2.3.8 Blood Pressure Measurement

After filling out about two‐thirds of the questionnaire and taking a break for around 40 min, the SBP and DBP were checked using a calibrated sphygmomanometer while sitting still, and three readings were taken at 5‐min intervals. The average of the second and third readings was used to determine the blood pressure [27].

#### 2.3.9 Measurement of Biochemical Factors

The Hazrat Ali Health Center's Laboratory gathered blood samples from participants after 12 h of fasting to measure HDL‐C, TG, and FBS levels. Participants were asked to avoid strenuous exercise, alcohol, and caffeinated beverages 24 h before the blood draw. The laboratory tests were conducted using an auto‐analyzer and a specific protocol.

#### 2.3.10 Dietary Assessment

The subjects were asked to record what they eat for 3 days per month. One day off and two workdays every month on the 10th, 18th, 25th, 38th, 50th, 55th, 69th, 73rd, and 19th days. Then, we used Nutritionist IV software version 3.5.2 to analyze the dietary information and recorded the amount of calorie and nutrients consumed [29].

#### 2.3.11 Assessment of Physical Activity

Each individual's daily PA record transformed into a metabolic equivalent [30, 31]. We asked that subjects maintain their usual PA routines without providing any specific guidance on how much PA to engage in.

#### 2.3.12 Intervention

Our study took place from July 26th, 2023 to October 26th, 2023. One group received the MIND diet and placebo, while the other group received the propolis and MIND diet for comparison. The placebo was a microcrystalline cellulose, and both groups followed regular food recommendations without any calorie restrictions being considered. The placebo was microcrystalline cellulose because it is a insoluble, nonreactive, neutral, white, and free‐flowing versatile excipient [32]. The placebo and the supplements were made at Mashhad University of Medical Sciences' Pharmacology Department, and both the researchers and the patients were unaware of what they were receiving until the study's conclusion. This was done to prevent any bias in the data collection process by having a different person deliver the intervention. Individually for each of the two groups, the initial visit involved a short conversation about the fundamentals of diet, recommendations for regular dietary guidance, and providing supplements.

Following the categorization, individuals who were enlisted worked alongside the researchers for a period of 3 months. The patients were required to keep a record of their food intake for at least 3 days each month, including 2 weekdays and 1 weekend day. They also had to contact us in person during the final week to ensure they were following the plan and by phone once a week. To qualify, participants needed to complete at least 70% of their dietary records (i.e., 2 out of 3 records) and were given guidance on how to monitor their diet daily. Additionally, motivational strategies like website activities and newsletters and were used to keep participants engaged.

#### 2.3.13 MIND Diet Group

The counseling plan for diet included instructions on which foods to eat and how to incorporate them into their diet.

The MIND diet is based on Morris's research. Those in the MIND group were taught how to modify their eating habits, focusing on incorporating natural and plant‐based foods, as well as consuming berries, olive oil, nuts, green leafy vegetables, whole grains, and fish while limiting intake of animal products and saturated fats. Due to Islamic culture and traditions, Iranians are prohibited from consuming alcohol. As a result, we advised our patients to substitute alcohol with grapes, grape juice, and raisins [33]. Although wine contains resveratrol, a polyphenol with potential neuroprotective effects, its consumption is not permissible in this population. Grapes and related products were therefore chosen as culturally acceptable substitutes that provide similar polyphenolic compounds and other beneficial nutrients, largely preserving the antioxidant and neuroprotective effects of wine while avoiding alcohol intake compounds such as resveratrol, anthocyanins, catechins, and flavonols, which exhibit antioxidant and anti‐inflammatory properties [34]. These compounds contribute to neuroprotective effects, support cerebral blood flow, and help prevent neuronal damage [35]. Additionally, both wine and grapes can positively influence metabolic health by improving insulin sensitivity, regulating blood glucose, and modulating lipid profiles, which is particularly beneficial for individuals with metabolic syndrome. They also help reduce oxidative stress, lower inflammation, and support cardiovascular health through improved endothelial function and reduced blood pressure [36]. Furthermore, grapes and wine provide essential nutrients such as vitamins C and K, potassium, and dietary fiber (mainly in grapes) [37]. Participants were instructed to go to the designated center three times, on days 7, 46, and 81. They were provided with the MIND diet and received counseling during their visits, which lasted at least 30 min. The patients′ adherence to the diet was monitored and they were offered counseling during each meeting, which lasted at least 15 min [38]. Additionally, they were provided with general dietary guidance, including written and verbal data about healthy food choices based on the healthy food plate. All participants were introduced to the fundamental principles of these food recommendations.

a. Take your time to savor each bite and chew it well while eating slowly.
b. Opt for grilled, steamed, or boiled foods instead of fried ones.
c. Before cooking or meat chicken, make sure to remove any excess skin and fat.
d. Choose mixed rice and oats and whole‐meal bread, over simple pasta and rice.
e. Please do not skip any meals.
f. Eat small meals frequently.
g. Cut down on sugary and fatty foods.

This mix of food aligns better with the eating habits of Iranians [29].

This group received a placebo as well.

The subjects were instructed on how to follow a specific diet independently. The study assessed compliance with the dietary intervention on a weekly basis using the MIND diet score questionnaire and food records. Participants who fulfilled 80% or more of their snacks and meals were considered to be adhering to the MIND diet [33].

#### 2.3.14 MIND Diet + Propolis Group

The MIND diet plus propolis group received the regular dietary advices, along with 450 mg of propolis to be taken twice daily before dinner and lunch [39] for 3 months. The dosage of propolis is decided by selecting a phase II trial, as mentioned in the study by Zhao et al. in 2016 [40].

#### 2.3.15 Compliance of the Subjects

The study involved three key points: the start, middle, and end, all focused on the participants. Participants were also monitored through weekly texts and phone calls to ensure they were taking the supplement regularly. They were also required to record their daily consumption on a table. Finally, the study included calculating the amount of unused supplement packaging returned by the participants at the end.

#### 2.3.16 Study Registration

**Figure 2:**
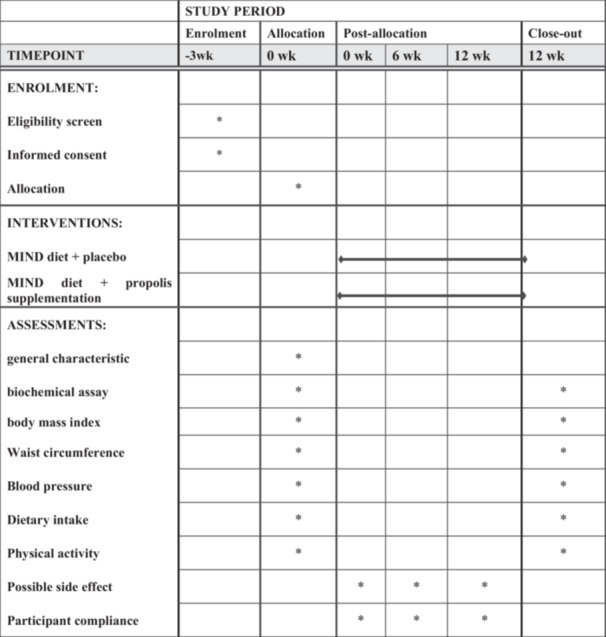
The SPIRIT figure details the schedule for assessments, interventions, and enrollment based on the Standard Protocol Items: Recommendations for Interventional Trials.

The current study′s procedure received approval from the Iranian Registry of Clinical Trials (www.irct.ir) on March 28, 2023, with the registration reference IRCT20230105057054N1. The study followed the Standard Protocol Items: Recommendations for Interventional Trials (SPIRIT) statement in its design and reporting. The SPIRIT diagram displayed in Figure 2 summarizes the schedule of participant enrollment, interventions, and evaluations.

#### 2.3.17 Sample Size and Sampling Method

$$n_{1}=\frac{({z_{1}+z_{1})}^{2}\;*\;2\;s^{2}}{d^{2}}+\frac{z_{1}^{\begin{array}{l} 2\\ \; \end{array}}}{4}$$

*z*_1_ = 95% confidence interval (CI) of the study = 1.96
*z*_2_ = 80% of the study focuses on power factor = 0.84
*s* = Estimate of SD = 16.7 = (100‐0) 1/6
*d* = to estimate the minimum average difference between two groups out of the two studied groups that make the difference meaningful = 13 [41, 42].

Sample size including 5% missing data For each group: *n* = 28 [41, 42].

#### 2.3.18 Information Analysis Methods and Tools

The data collected from participants will be analyzed at both descriptive and analytical levels. Descriptive analysis will include statistical summary measures, such as indices of central tendency and dispersion, and will be presented using tables and graphs. Analytical procedures will consist of a primary analysis, including paired *t*‐tests and analysis of variance, followed by a final analysis employing a generalized linear model (GLM) with a Gamma distribution and log link function to adjust for potential confounding factors: the influence for drug, baseline, PA, education, food intake, hospitalization, supplementation, gender, surgery, and age. we only used the average and ANOVA for reporting as usual. Considering this problem, we used GLM with distribution of Gamma and link of log for the final analysis and then we concluded. We did not judge by the average, we judged by the data rank, which is a change from the mean. This approach was chosen because the outcome variables were continuous but not normally distributed. Instead of using parametric methods such as ANCOVA, which assume normality, the GLM with Gamma distribution served as an appropriate alternative. A GLM with a gamma distribution and a log link function was used to analyze the outcomes. The gamma distribution approximates normality with a sufficiently large sample size. Since the study included two groups and some variables were slightly non‐normally distributed, non‐parametric tests would typically be required. Using the GLM provides a parametric alternative to ANCOVA, allowing adjustment for baseline covariates while accommodating non‐normal outcome distributions [43, 44].

We checked if the distribution of quantitative variables was normal using the Shapiro‐Wilk test and by looking at skewness. We provided the mean SD or median [interquartile range] for quantitative variables and used numbers (percentages) for qualitative variables. For each outcome, we showed the estimated effect size and its 95 percent CI. For comparison, we used Chi‐square tests, with all tests being two‐tailed and having a significance level of 0.05. The Kruskal–Wallis test was employed to determine the crude p‐values. Statistical reporting adhered to the guidelines of the Statistical Analyses and Methods in the Published Literature (SAMPL) framework [45]. All analyses were performed using SPSS Statistics software, version 26.

## 3 Results

This study recruited 56 subjects between the ages of 18 and 60. They were divided into two groups, one receiving the MIND diet with propolis and the other receiving the MIND diet with a placebo, with 28 people in each group. We maintained regular communication with all participants to ensure complete data collection; Continuous phone contact was maintained with each participant throughout the study. If a participant did not respond, multiple attempts were made to reach them. As a result, there were no missing data after starting intervention in this study, and all 56 participants completed the study without dropping out. There were no significant differences in PA, food intake, education, age, gender, surgery, drug use, supplementation, or hospitalization between the two groups (*p*‐value > 0.05). The general characteristic of patients is shown in Table 1.

**Table 1:** Baseline demographic information of the study patients (*n *= 56).

Variable		Baseline		
		Mind‐ propolis	Mind‐placebo	*p*‐value
Age; mean ± SD	Year	52.89 (6.5)	51.32 (5.3)	0.165
Physical Activity; mean ± SD	Met‐min/wk	845.8 (641.7)	1034.4 (1195.2)	0.843
Food intake; mean ± SD	Kcal/day	1496.7 (504.6)	1379.8 (392.6)	0.205
Gender; N (%)	Male	9 (32.1)	13 (46.4)	
	Female	19 (67.9)	15 (53.6)	0.274
Education; N (%)	≥ diploma	14 (50.0)	16 (57.1)	0.592
	> diploma	14 (50.0)	12 (42.9)	
Hospitalization; N (%)	No	17 (60.7)	17 (60.7)	1.000
	Yes	11 (39.3)	11 (39.3)	
Surgery; N (%)	No	14 (50.0)	16 (57.1)	0.592
	Yes	14 (50.0)	12 (42.9)	
Drug; N (%)	No	20 (71.4)	18 (64.3)	0.567
	Yes	8 (28.6)	10 (35.7)	
Supplementation; N (%)	No	18 (64.3)	15 (53.6)	0.415
	Yes	10 (35.7)	13 (46.4)	

Abbreviations: Kcal, kilocalorie; MET, metabolic equivalent task; min, minute; N, number; SD, standard deviation; wk, week.

Mean before BMI, DBP, SBP, WC, weight, HDL‐C, TG, and FBS hadn′t significant difference at baseline (*p*‐value > 0.05). All MetS indices showed a nonsignificant decrease after the intervention, except for FBS which significantly decreased, while HDL‐C showed a nonsignificant increase. Table 2 is showed the MetS indices.

**Table 2:** Metabolic syndrome indices of the study patients before and after the intervention (*n *= 56).

Variable	Before			After		
	Mind‐propolis‐1 (mean + SD)	MIND‐placebo‐2 (mean + SD)	Crud‐ *p*‐value*	Mind‐propolis‐1 (mean + SD)	MIND‐placebo‐2 (mean + SD)	Crud‐ *p*‐value*
Weight (Kg)	76.4 ± 10.8	80.8 ± 11.8	0.121	74.8 ± 11.1	80.02 ± 11.9	0.125
BMI (Kg/m^2)^	29.2 ± 4.3	29.8 ± 5.3	0.594	28.6 ± 4.5	29.5 ± 5.2	0.441
WC (Cm)	98.1 ± 8.5	99.1 ± 8.8	0.640	96.6 ± 8.5	97.2 ± 8.3	0.749
SBP (mmHg)	125.6 ± 14.06	123.7 ± 12.9	0.517	118.3 ± 13.8	119.7 ± 11.2	0.850
DBP (mmHg)	79.6 ± 8.0	79.8 ± 10.3	0.831	75.9 ± 8.4	75.6 ± 9.6	0.792
MAP (mmHg)	94.9 ± 9.1	94.5 ± 10.0	0.863	90.00 ± 9.00	90.3 ± 8.9	0.670
FBS (m g/dL)	104.4 ± 14.8	113.3 ± 12.5	0.066	99.4 ± 16.4	105.5 ± 11.8	0.029
TG (m g/dL)	201.1 ± 84.8	195.3 ± 95.9	0.583	157.7 ± 54.5	143.2 ± 65.6	0.159
HDL‐C (mg/dL)	39.4 ± 9.5	36.04 ± 4.7	0.137	46.3 ± 7.9	45.1 ± 6.9	0.328
MIND‐score (0‐14)	7.04 ± 1.6	7.2 ± 2.0	0.895	12.2 ± 1.3	12.4 ± 1.03	0.697

Abbreviations: BMI, body mass index; DBP, diastolic blood pressure; FBS, fasting blood sugar; HDL‐C, high‐density lipoprotein cholesterol; MIND, Mediterranean‐DASH Intervention for Neurodegenerative Delay; SBP, systolic blood pressure; SD, standard deviation; TG, triglyceride; WC, waist circumference.

* Using Kruskal–Wallis test.

Initially, there was significant difference in dietary intake between the two groups. MIND score was 7.04 ± 1.6 and 7.2 ± 2.0 in MIND + propolis and MIND groups, respectively, and there was nonsignificant difference between groups. MIND score significantly increases to 12.4 ± 1.03 (*p* < 0.001) and 12.2 ± 1.3 (*p* < 0.001) in the MIND group and the MIND + Propolis group, respectively. The patients were adhered to the dietary compound consumption which was insignificant for all items (in the MIND + propolis and MIND groups) (Table 3). There wasn′t significant difference in dietary intake between groups after consumption. Providing dietary guidelines alone is not sufficient; the effectiveness of a diet depends on participants′ adherence to it. Table 3 on adherence to the MIND diet demonstrates whether participants actually followed the diet and whether the observed effects in the outcomes can be attributed to the intervention. Without assessing adherence, the relationship between the diet and changes in MetS indices cannot be definitively interpreted. Therefore, adherence is a key indicator of study quality and the validity of the results. Table 3 demonstrates how well the two groups followed the MIND diet before and after the study.

**Table 3:** Adherence to mind diet in two groups.

Variable		Before			After		
		MIND‐ propolis	MIND‐Placebo	*p*‐value	MIND‐ propolis	MIND‐Placebo	*p*‐value
Green vegetable	≥ 2 s/w	13 (46.4)	12 (42.9)	0.641	0 (0.0)	0 (0.0)	0.305
	2–6/w	9 (32.1)	7 (25.0)		3 (10.7)	1 (3.6)	
	≥ 6 s/w	6 (21.4)	9 (32.1)		25 (89.3)	27 (96.4)	
Vegetable	≥ 5 s/w	13 (46.4)	9 (32.1)	0.547	0 (0.0)	0 (0.0)	0.500
	5‐7/w	3 (10.7)	5 (17.9)		1 (3.6)	0 (0.0)	
	≥ 1 s/d	12 (42.9)	14 (50.0)		27 (96.4)	28 (100)	
Berry	≥ 1 s/w	21 (75.0)	23 (82.1)	0.257	6 (21.4)	2 (7.1)	0.344
	1‐2/w	6 (21.4)	2 (7.1)		2 (7.1)	1 (3.6)	
	≥ 2 s/w	1 (3.6)	3 (10.7)		20 (71.4)	25 (89.3)	
Nuts	< 1/m	6 (21.4)	11 (39.3)	0.300	1 (3.6)	1 (3.6)	0.741
	1/m to < 5/w	11 (39.3)	10 (35.7)		0 (0.0)	2 (7.1)	
	≥ 5 s/w	11 (39.3)	7 (25.0)		27 (96.4)	25 (89.3)	
Olive oil	Not primary oi**l**	22 (78.6)	23 (82.1)	0.737	7 (25.0)	6 (21.4)	0.752
	primary oil used	6 (21.4)	5 (17.9)		21 (75.0)	22 (78.6)	
Butter	> 2 tsp/d	3 (10.7)	5 (17.9)	0.789	0 (0.0)	0 (0.0)	0.500
	1‐2 tsp/d	5 (17.9)	5 (17.9)		2 (7.1)	3 (10.7)	
	< 1 tsp/d	20 (71.4)	18 (64.3)		26 (92.9)	25 (89.3)	
Cheese	≥ 7 s/w	**10 (35.7)**	5 (17.9)	0.200	2 (7.1)	2 (7.1)	1.000
	1‐7/w	15 (53.6)	22 (78.6)		17 (60.7)	16 (57.1)	
	< 1 s/w	3 (10.7)	1 (3.6)		9 (32.1)	10 (35.7)	
Wholegrains	< 1 s/d	16 (57.1)	12 (42.9)	0.558	0 (0.0)	0 (0.0)	0.500
	1–3/d	10 (35.7)	14 (50.0)		2 (7.1)	3 (10.7)	
	≥ 3 s/d	2 (7.1)	2 (7.1)		26 (92.9)	25 (89.3)	
Fish	Rarely	19 (67.9)	16 (57.1)	0.476	7 (25.0)	5 (17.9)	0.747
	1–3/m	4 (14.3)	8 (28.6)		7 (25.0)	9 (32.1)	
	≥ 1 meals/w	5 (17.9)	4 (14.3)		14 (50.0)	14 (50.0)	
Bean	< 1 meals/w	10 (35.7)	12 (42.9)	0.515	1 (3.6)	2 (7.1)	1.000
	1–3/w	16 (57.1)	12 (42.9)		6 (21.4)	5 (17.9)	
	> 3 meals/w	2 (7.1)	4 (14.3)		21 (75.0)	21 (75.0)	
Poultry	< 1 meal/w	8 (28.6)	6 (21.4)	0.776	2 (7.1)	2 (7.1)	0.373
	1–2/w	8 (28.6)	10 (35.7)		3 (10.7)	7 (25.0)	
	≥ 2 meals/w	12 (42.9)	12 (42.9)		23 (82.1)	19 (67.9)	
Meat	> 6 meal/w	0 (0.0)	0 (0.0)	0.695	0 (0.0)	0 (0.0)	‐‐‐
	4–6/w	2 (7.1)	2 (7.1)		0 (0.0)	0 (0.0)	
	< 4 meals/w	26 (92.9)	26 (92.9)		28 (100)	28 (100)	
Fast‐food	≥ 4 times/w	0 (0.0)	1 (3.6)	1.000	0 (0.0)	0 (0.0)	0.500
	1–4/w	5 (17.9)	4 (14.3)		2 (7.1)	1 (3.6)	
	< 1 time/w	23 (82.1)	23 (82.1)		26 (92.9)	27 (96.4)	
Sweet	≥ 7 s/w	2 (7.1)	2 (7.1)	0.911	0 (0.0)	0 (0.0)	0.500
	5–7/w	10 (35.7)	8 (28.6)		3 (10.7)	2 (7.1)	
	< 5 s/w	16 (57.1)	18 (64.3)		25 (89.3)	26 (92.9)	

Abbreviations: d, day; m, month; N, number; s, serving; tsp, teaspoon; w, week.

MIND plus propolis group after adjusting variables showed a nonsignificant reduce (*p*‐value > 0.05) about BMI (Kg/m^2^) by 0.8 times (20%), and showed a nonsignificant reduce about TG (mg/dL) by 0.9 times (10%), and nonsignificant increase about WC (cm) and MAP (mmHg) by 1.03 times (3%) and 1.3 times (30%), respectively, and HDL‐C (mg/dL) has shown a significant increase by 12.8 times (Table 4).

**Table 4:** MIND diet and propolis on difference of syndrome metabolic indices.

Variable		Exp (B)^a^	*p*‐value
Diff‐BMI	Kg/m^2^	0.8	0.374
Diff‐WC	Cm	1.03	0.874
Diff‐Map	mmHg	1.3	0.285
Diff‐FBS	Mg/dL	1.0	0.960
Diff‐TG	Mg/dL	0.9	0.685
Diff‐HDL	Mg/dL	12.8	< 0.001

Abbreviations: B, regression coefficient; BMI, body mass index; Diff, difference; Exp, exponential; FBS, fasting blood sugar; HDL, high‐density lipoprotein cholesterol; MAP, mean arterial pressure; TG, triglyceride; WC, waist circumference.

^a^ Using generalized linear model (GLM) with distribution of Gamma & link of Log controlling for baseline, food intake, PA, hospitalization, education, supplementation, drug, gender, surgery, and age.

This study did not report any serious adverse effects or toxicity.

## 4 Discussion

This study indicated that MIND score significantly increases in the MIND group and the MIND + Propolis group. The patients were adhered to the dietary compound consumption which was insignificant for all items (in the MIND plus propolis and MIND groups). There wasn′t significant difference in dietary intake between groups after consumption. MIND + propolis group compared to MIND group showed nonsignificant decrease about BMI after adjusting variables, and showed nonsignificant decrease about TG, and nonsignificant increase about WC and MAP, and HDL‐C has shown a significant increase. This study did not report any serious adverse effects or toxicity.

Propolis is a substance collected from plant buds and secretions by bees, and it is then processed with saliva and bee enzymes to become a compound and adhesive material used within the hive [10, 11]. In recent years, propolis has garnered significant interest from researchers because of its various biological and medicinal properties, including its ability to modulate the immune system, its antitumor effects, its antimicrobial and anti‐inflammatory properties, as well as its antioxidant activity [12]. Propolis extract improves memory performance and helps regulate social behavior, protecting the prefrontal cortex and hippocampus from damage in adult male rats [46]. Supplementation with *Euphorbia resinifera* propolis enhanced memory in rats fed a high‐fructose diet. Its antioxidant properties likely contribute to this effect and support its traditional use in Morocco. Therefore, *Euphorbia resinifera* propolis may have potential as a therapeutic agent for memory impairments induced by high‐fructose intake [47]. Several investigations have indicated a notable decrease in serum lipid profiles, fasting glucose levels, insulin resistance, insulin, HbA1c, interleukin‐6, TNF‐α, CRP, and liver enzymes aspartate aminotransferase, alanine transaminase after consumption of propolis [10, 11] and increase HDL‐C [11]. Another study revealed an elevation in HDL‐C and a notable reduction in TG. Nevertheless, there were no substantial alterations noted for LDL‐C, total cholesterol, BMI, and body weight [13]. In subjects with central obesity, propolis can reduce leptin levels [14]. One meta‐analysis study indicated a noteworthy decrease in FBS following propolis intake [15].

A review study demonstrated the potential efficacy of propolis in controlling MetS and its associated markers, possibly due to its substantial anti‐inflammatory and antioxidant properties [16]. Evidence from a review article indicates that honey bee propolis contributes to the enhancement of body cell function and the reduction of diabetes‐related complications [17]. The recent study showed that propolis may help reduce waist circumference and enhance physical health and quality of life in people with this syndrome, but it did not have any notable impact on other component of MetS [18]. propolis Polyphenols have been shown to influence thermogenesis, browning, and lipid metabolism, potentially playing a role in body weight regulation [19].

Consistent with our findings and Yu Yang et al. [48], Verónica Mujica et al. [49] indicated that Propolis may have a positive impact on HDL‐C. In contrast to our findings, Samadi et al. [50] reported that propolis supplementation did not significantly affect HDL levels. The effect of propolis on lipid profiles is thought to be due to its potential to enhance the expression of Adenosine triphosphate (ATP)‐binding cassette transporters in liver proteins. These transporters are associated with the production of HDL‐C and the removal of lipids from peripheral tissues, which could explain the beneficial effects of propolis on lipid levels [48, 50]. On the other hand, propolis might boost the activation of the ATP‐binding cassette transporter A1 (ABCA1) pathway by encouraging the expression of Peroxisome Proliferator‐Activated Receptor (PPAR) gamma and liver X receptor. This encouragement can lead to a reduction in cholesterol buildup in macrophage foam cells [51]. Propolis decreases blood sugar that may be due to its bioactive components, that could help increase insulin production and improve the body′s response to insulin [49]. Propolis may have the ability to improve the absorption of glucose and the activity of insulin‐sensitive glucose transporter (GLUT) 4 in muscle cells by activating phosphatidylinositol 3‐kinase (PI3K) and monophosphate‐activated protein kinase (AMPK) phosphorylation, which can help increase cellular glucose use [52]. Additionally, propolis has the ability to inhibit the function of genes related to gluconeogenesis, specifically glucose‐6‐phosphatase, in liver cells [53]. Propolis appears to slow down the development and manifestation of MetS by working through three mechanisms: inhibiting the expression of advanced glycation end products (AGEs) and their receptors (RAGEs), decreasing pro‐inflammatory signaling pathways, and improving the cellular antioxidant systems [16].

Consistent with our findings in multiple review studies, pooled analyses of propolis supplementation showed no significant impact on body anthropometric measures [10, 13]. Overall, several human RCTs to date have similarly reported that propolis has little or no effect on weight and BMI [11, 49]. Consistent with our findings, Liting Zhao et al. [40], Weina Gao et al. [54], Takuya Fukuda [55] reported that Brazilian Green propolis supplementation did not have a significant effect on blood glucose levels in Patients with Type 2 Diabetes Mellitus. Consistent with our findings in several meta‐analysis studies, propolis supplementation showed no significant impact on TG [10, 56]. Consistent with our findings in several meta‐analysis studies, propolis supplementation showed no significant impact on DBP [57, 58]. Although propolis has been reported to exert antioxidant, anti‐inflammatory, and lipid‐modulating effects, our study did not demonstrate significant reductions in TG, WC, SBP, or DBP. This lack of significant changes may be attributed to the relatively short intervention duration, the administered dose, or baseline characteristics of the participants, such as degree of adiposity and metabolic status. Mechanistically, propolis is thought to reduce oxidative stress, inhibit pro‐inflammatory cytokines, and improve lipid metabolism by modulating key enzymes involved in cholesterol and triglyceride synthesis. However, these effects may require longer exposure or higher doses to translate into measurable improvements in clinical parameters. These findings highlight the need for further long‐term, adequately powered studies to fully elucidate the metabolic benefits of propolis.

### 4.1 Strengths and Limitations

The study has several strengths. (a) This is the initial study to assess how MIND diet + propolis affects MetS indicators among using a RCT, (b) the study controlled for confounding factors such as baseline characteristics, education, age, surgery, gender, hospitalization, supplementation, drug, food intake, and PA, (c) Blood samples were taken at the health center where patients were recruited. However, there are also limitations in the current study: (a) because of budget limitations, (b) some factors including inflammatory biomarkers and antioxidants (e.g., CRP, TNF‐α) could not be measured. (c) the duration of the study was relatively short which may have had significant effects on the results (d) a large sample size.

## 5 Conclusion

Propolis supplementation resulted in a nonsignificant decrease in BMI and TG, a nonsignificant increase in WC and MAP, and a significant increase in HDL‐C. Additionally, participants reported subjective improvements in constipation and related symptoms following propolis use. These findings suggest potential benefits of propolis on metabolic parameters and gastrointestinal health. We recommend further studies to confirm these effects and to explore the clinical relevance of propolis supplementation in patients with metabolic syndrome.

## Author Contributions

**Zeinab Gholami:** conceptualization, formal analysis, writing – original draft, resources, project administration, validation, investigation, data curation, software, methodology. **Mohammad Reza Maracy:** validation, formal analysis, writing – review and editing. **Mohammad Reza Abbaspoor:** resources, writing – review and editing. **Zamzam Paknahad:** writing – review and editing, conceptualization, funding acquisition, supervision, validation, visualization.

## Author Declaration

The lead author Zamzam Paknahad affirms that this manuscript is an honest, accurate, and transparent account of the study being reported; that no important aspects of the study have been omitted; and that any discrepancies from the study as planned (and, if relevant, registered) have been explained. All authors have read and approved the final version of the manuscript, Z.P. had full access to all of the data in this study and takes complete responsibility for the integrity of the data and the accuracy of the data analysis.

## Ethics Statement

The current study complies with the Declaration of Helsinki and its subsequent revisions. It has been approved by the Medical Ethics Committee of Isfahan University of Medical Sciences with the ethics code IR.MUI.RESEARCH.REC.1401.330 and grant number 3401567. Before participating, all participants provided verbal and written informed consent. The protocol for the study was approved by the Iranian Registry of Clinical Trials (www.irct.ir) with registration reference IRCT20230105057054N1 on March 28, 2023.

## Consent

The authors have nothing to report.

## Conflicts of Interest

The authors have stated that they do not have any conflicts of interest. They also confirm that the funding source or financial relationships will not be involved in the report writing, data interpretation, analysis, data collection, the decision to publish the report, or study design. There are no declarations of interest.

## Supporting information

consort diagram.

## Data Availability

Zamzam Paknahad and all authors have read and approved the final version of the manuscript have full access to all of the data in this study and take complete responsibility for the integrity of the data and the accuracy of the data analysis. The datasets generated and/or analyzed during the current study are not publicly available due to some restrictions applied by the ethics committee; but are available from the corresponding author on reasonable request. You can get the questionnaires in Persian if you ask for them. The data that support the findings of this study are available on request from the corresponding author. The data are not publicly available due to privacy or ethical restrictions.
