# Supplementary material for: Effects of Propolis Supplementation on Metabolic Syndrome Indices, Isfahan, Iran, 2023: A Randomized Controlled Clinical Trial
Source: Health Sci Rep. 2025 Nov 13;8(11):e71499. doi: 10.1002/hsr2.71499 (PMC12613288; doi:10.1002/hsr2.71499)
Supplement: Supplementary file 1 — consort diagram. [file HSR2-8-e71499-s001.docx]

Assessed eligibility (n=2000)

Excluded (n=355)

Not meeting inclusion criteria (n= 563)

Declined to participate (n=897)

Other reasons (n=125)

Enrolment

Randomized (n=60)

Allocated to intervention (MIND) (n=28)

Received allocated intervention (n=28)

Did not receive allocated intervention (give reasons (n=2) one individuals withdrew

one participant has initiated another diet

Allocated to intervention (MIND + propolis) (n=28)

Received allocated intervention (n=28)

Did not receive allocated intervention (give reasons (n=2) two individuals withdrew

Allocation

Analysed (n=28)

Excluded from analysis (give reasons) (n=0)

Lost to follow-up (give reasons) (n=0)

Discontinued intervention (give reasons) (n=0)

Follow-up

Lost to follow-up (give reasons) (n=0)

Discontinued intervention (give reasons) (n=0)

Analysed (n=28)

Excluded from analysis (give reasons) (n=0)

Analysis
